# Gender-Specific DNA Methylation Profiles Associated with Adult Weight in Hezuo Pigs

**DOI:** 10.3390/ijms252111488

**Published:** 2024-10-25

**Authors:** Rui Jia, Xiaoyu Huang, Jiaojiao Yang, Longlong Wang, Jie Li, Yao Li, Shuangbao Gun, Zunqiang Yan, Pengfei Wang, Qiaoli Yang

**Affiliations:** 1 College of Animal Science and Technology, Gansu Agricultural University, Lanzhou 730070, China; jiaruirui0920@163.com (R.J.); huangxy@gsau.edu.cn (X.H.); work520819@126.com (J.Y.); wanglonglong1222@163.com (L.W.); lijie5272@126.com (J.L.); ly926111@163.com (Y.L.); gunsb@gsau.edu.cn (S.G.); yanzunqiang@163.com (Z.Y.); wangpf815@163.com (P.W.); 2 Gansu Innovations Center for Swine Production Engineering and Technology, Lanzhou 730070, China

**Keywords:** Hezuo pigs, DNA methylation, whole genome bisulfite sequencing, weight

## Abstract

The Hezuo pig, an important native Tibetan breed in China, exhibits differences in adult body weight, with females typically heavier than males. The underlying mechanisms for this disparity remain unclear. DNA methylation changes are known to influence animal growth and development and regulate Hezuo pig growth by altering gene expression related to these processes, thus differentially affecting adult body weight between genders. This study conducted DNA methylation analysis and expression profiling using pituitary tissues from male and female Hezuo pigs at 3 and 8 months old (M3M, M3F, M8M, and M8F). In total 346, 795, 371, and 839 differentially methylated genes (DMGs) were identified in the M3M vs. M3F, M3F vs. M8F, M3M vs. M8M, and M8M vs. M8F groups, respectively. The comparative analysis of differentially methylated regions (DMRs) genes and DEGs (differentially expressed regions) revealed that key genes involved in growth, hormone secretion, and the hypothalamic-pituitary-gonadal axis are primarily enriched in signaling pathways such as PI3K-Akt, Hippo, and adrenergic. Further analysis combining methylation and transcriptomics identified five candidate methylated genes (*CCL2*, *MYL2*, *GST*, *CTSH*, and *MCH*) linked to adult body weight in Hezuo pigs. Additionally, the correlation analysis suggested that these genes influence growth and development in boars and sows by regulating the secretion and synthesis of related hormones, leading to heavier weights in females. In conclusion, variations in adult body weight between male and female pigs may stem from the impact of DNA methylation on gene expression related to growth and development. These findings offer new insights into the regulatory mechanisms of DNA methylation during weight gain in Hezuo pigs.

## 1. Introduction

Differences in growth and development exist between males and females in many animal species. This leads to one sex being larger or growing faster than the other. Factors such as body weight, physiognomy, and form differ among species. Throughout biological evolution, males in many species have developed adaptations for successful mating. They exhibit exaggerated traits like stunning feathers, strong bodies, and large horns; these result from sexual selection. Typically, body weight is a significant phenotype in mammals, with males usually outweighing females. However, exceptions include species like Aucha Perch, Domestic Pig, Mantis, Blue Whale, and Goshawk, where females are heavier. In certain mammals, size differences between males and females can vary. In Aucha Perch, females can weigh up to 1 kg more than males under similar conditions. However, beyond this weight threshold, females tend to be smaller [1]. This disparity may arise because during their sexual cycle, Aucha Perch allocate a greater portion of consumed nutrients towards gonadal development rather than body weight gain. Conversely, Aucha Perch utilize fewer nutrients for gonadal development, facilitating faster body weight gain. Additionally, certain mammals display the phenomenon of larger females and smaller males, including miniature pigs and *Mus musculus*.

Pigs serve as primary meat animals. Foreign pig breeds typically feature males heavier than females, whereas certain domestic breeds display the reverse trend. Examples include the Diqing Tibetan pig, the Bama miniature pig, the small fragrant pig, and the Hezuo pig. Research shows that adult Bama miniature pigs weigh 39.66 kg for females and 34.06 kg for males [2]. Hezuo pigs, significant among China’s plateau Tibetan breeds, thrive in the Gannan Tibetan Autonomous Prefecture in Xiahe, Zhuoni, and Diebu. These pigs have superior meat quality and resilience [3,4]. Hezuo pigs raised semi-grazed in mixed agricultural-pastoral areas have lower nutritional requirements and growth rates compared to other local breeds. From 30 days old, they graze daily on plant roots and stems. At night, after herding, only minimal supplements like soybean residue and alfalfa grass pellets are provided. This extensive feeding continues throughout their lives. Hezuo pigs reach sexual maturity early; boars show sexual interest by 45 days and mature by 3 to 4 months. Sows first enter estrus at 4 to 5 months, with an estrous cycle of 18 days lasting 3 to 4 days. Studies on growth patterns reveal that males and females weigh similarly up to 150 days, but females significantly outgain males by 180 days (31.40 ± 1.41 kg vs. 30.20 ± 1.13 kg) and 210 days (37.0 ± 2.98 kg vs. 34.60 ± 3.12 kg) [5,6]. This weight disparity hinders meat production in male Hezuo pigs. The pituitary gland is a crucial endocrine organ. It regulates postnatal growth through hormonal control, particularly by secreting growth hormone (GH) and insulin-like growth factor-1 (IGF-1) to promote weight gain [7,8]. By focusing on the pituitary, we aim to gain insights into the epigenetic regulation of hormone synthesis and secretion and how these processes might be altered during development.

DNA methylation, a key epigenetic modification, regulates growth in animals [9,10,11]. This process can induce heritable changes in chromatin structure, DNA conformation, stability, and the interaction between DNA and proteins, thereby regulating gene expression [12]. Reports indicate that DNA methylation influences pig growth and development by regulating gene expression [7]. Thus, studying methylation is crucial for understanding body weight growth mechanisms in Hezuo pigs. Yet, the role of DNA methylation in the weight differences between female and male Hezuo pigs remains unclear.

In this research, we focused on boars and sows aged 3 months and 8 months as our experimental subjects. The 3-month-old pigs signify a crucial developmental phase marked by a notable surge in body weight as they undergo rapid growth. Our exploration of DNA methylation during this period seeks to clarify the epigenetic regulatory frameworks that facilitate this accelerated growth and development. On the other hand, the 8-month-old pigs were approaching maturity in terms of body size. At this stage, while their growth rate slowed, it continued to be positive, and as their body weight reaches a specific threshold, the rate of growth gradually diminishes. Investigating DNA methylation at this age will shed light on whether epigenetic alterations play a role in the transition from growth to maturity in Hezuo pigs and how these changes affect their eventual phenotypic characteristics. This study hypothesizes that dynamic DNA methylation changes modulate epigenetic variations in Hezuo pigs of different ages. We aimed to identify differentially methylated regions (DMRs) in pituitary tissues from 3- and 8-month-old Hezuo pigs and assess the relationship between transcriptome profiles and DNA methylation patterns. These findings offer new insights into the regulatory mechanisms of DNA methylation during weight gain in Hezuo pigs and provide references for studying methylation modifications related to animal sex traits.

## 2. Results

### 2.1. Changes in Serum Hormone Content in Male and Female Hezuo Pigs

**Figure 1:**
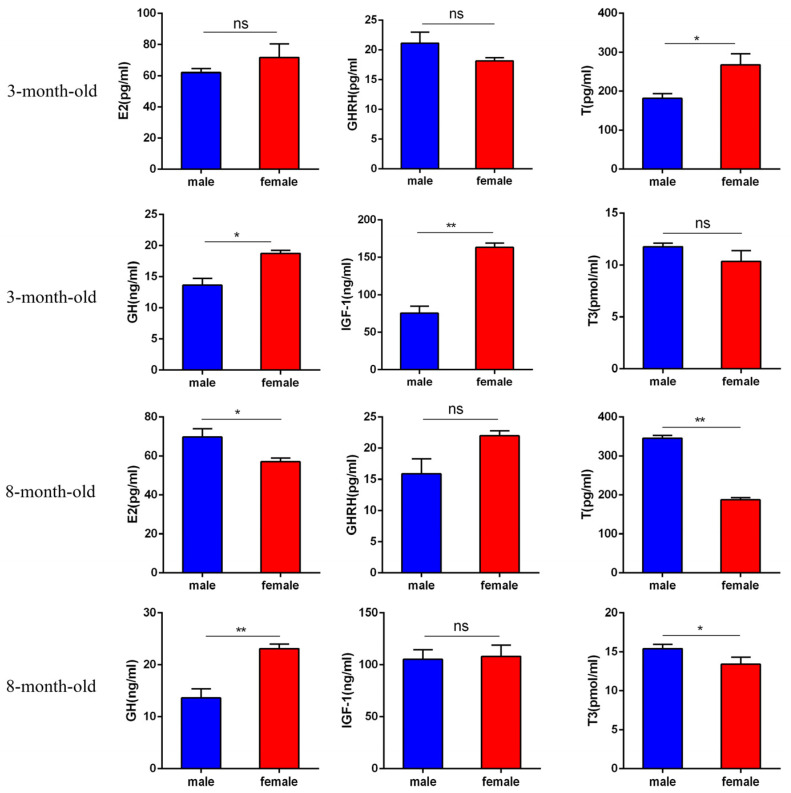
Differences in serum hormone content between 3- and 8-month-old male and female Hezuo pigs (** *p* < 0.01; * *p* < 0.05; ns means not significant).

As shown in Figure 1, 3-month-old Hezuo pigs had significantly higher serum levels of IGF-1 in females than in males (*p* < 0.01). Females also had higher serum levels of testosterone (T) and growth hormone (GH) than males (*p* < 0.05), while levels of estradiol (E2), growth hormone-releasing hormone (GHRH), and triiodothyronine (T3) showed no significant differences between the two genders (*p* > 0.05). In 8-month-old Hezuo pigs, females exhibited significantly higher serum GH levels than males (*p* < 0.01). Conversely, males had significantly higher serum levels of T, E2, and T3 than females (*p* < 0.01 for T and *p* < 0.05 for E2 and T3). Serum levels of GHRH and IGF-1 had no significant differences between genders.

### 2.2. Whole Genome Bisulfite Sequencing (WGBS) Sequencing of Pituitary Tissue in Male and Female Hezuo Pigs

WGBS sequencing was performed on pituitary tissues from M3M, M3F, M8M, and M8F Hezuo pigs. Approximately 1.87 billion high-quality clean reads were obtained, averaging 468 million reads per sample. The conversion rate was 99% (99.42% to 99.52%). An average of 83.19% (82.96% to 83.33%) of reads mapped to the pig reference genome (Sus scrofa 11.0), with an average sequence depth of 23.48. Sequencing data quality details are listed in Table 1. The results indicated high sequencing quality and successful bisulfite modification.

### 2.3. Sequencing Depth Statistics for Genome-Wide Methylated CG, CHG, and CHH

**Figure 2:**
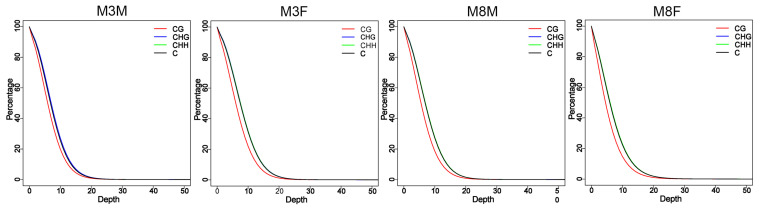
Sequencing depth map in the four-group base.

As shown in Figure 2, the cumulative distribution of methylated C bases in M3M, M3F, M8M, and M8F Hezuo pigs was similar at the same effective sequencing depths. Notably, with increased sequencing depth, the coverage of C bases decreased. Since the total number of C bases in CHH and CHG forms is small, the coverage trend remained consistent, aligning with that of CG.

### 2.4. Analysis of C-Base Methylation Level in Pituitary Tissue of Hezuo Pig

The whole genome methylation level reflects the overall genome methylation map characteristics. Data in Table 2 show the methylation levels of C bases in the pituitary tissue of Hezuo pigs. The methylation rates for C sites in the whole genome of M3M, M3F, M8M, and M8F were 5.15%, 4.92%, 4.97%, and 4.76%, respectively. The methylation rates for CG sites in M3M, M3F, M8M, and M8F were 76-77%, respectively. Methylation rates for CHG and CHH sites were about 1% across all four groups.

**Figure 3:**
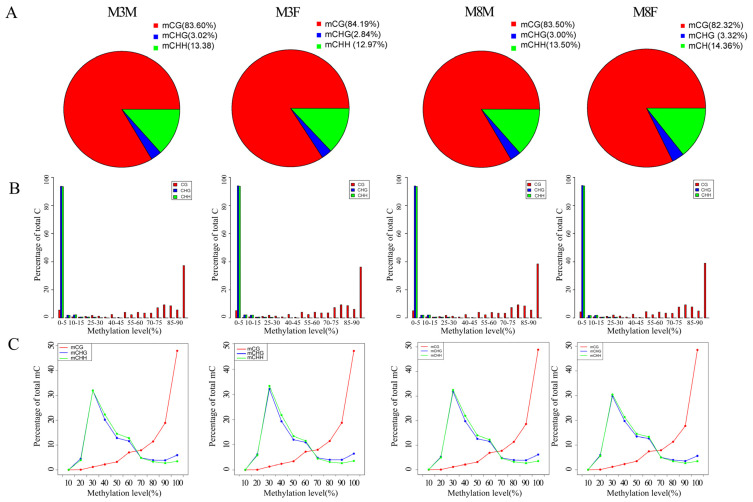
Analysis of C-base methylation levels: (**A**) Distribution of methylation C sites (mC) among the four groups for three DNA methylation patterns; (**B**) Different distribution types of C bases (CG, CHG, and CHH) among the four groups; (**C**) Genome-wide methylation levels among the four groups.

We identified and analyzed the distribution of methylated C sites (mC) in three DNA methylation patterns: mCG, mCHG, and mCHH (where H is A, C, or T). Genome-wide methylated cytosine (mC) accounted for 82.32% to 84.19% of CpG methylation, followed by CHH methylation (12.97% to 14.36%) and then CHG methylation (2.84% to 3.32%). This indicates that methylation in Hezuo pigs predominantly occurs at CG base sites, with similar proportions across groups (Figure 3A). The different distribution types of C bases (CG, CHG, and CHH) reflect the DNA methylation characteristics of Hezuo pigs. The methylation levels of C sites in various sequence environments in the pituitary tissue genome of M3M, M3F, M8M, and M8F Hezuo pigs were analyzed. The results showed that as methylation levels increased, CG sequences accounted for the highest proportion of all C sites (Figure 3B). Figure 3C shows that the proportion of CG in M3M, M3F, M8M, and M8F Hezuo pigs was significantly lower than that of CHG and CHH at low methylation levels. However, as methylation increased, the proportion of CG gradually exceeded that of CHG and CHH. The proportion of CG increased with rising methylation levels, whereas the proportions of CHG and CHH first increased and then decreased.

**Figure 4:**
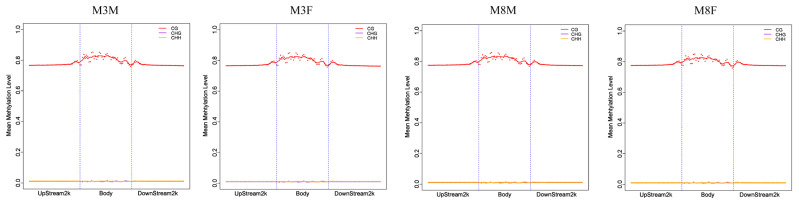
Distribution characteristics of DNA methylation levels in different functional regions among the four groups.

Analyzing the distribution characteristics of DNA methylation levels in various functional regions can assist in understanding the role of DNA methylation modification at the genome-wide level. In this study, DNA methylation distribution in the upstream 2 kb, genomic, and downstream 2 kb regions of the whole genome in pituitary tissue from M3M, M3F, M8M, and M8F Hezuo pigs was analyzed. The results indicated that across different genomic functional element regions, DNA methylation levels in M3M, M3F, M8M, and M8F Hezuo pigs were similar, with CG methylation levels being higher than those of CHG and CHH. Methylation levels in exons and introns at CG sites were higher than in the flanking regions with upstream 2 kb and downstream 2 kb (Figure 4).

**Figure 5:**
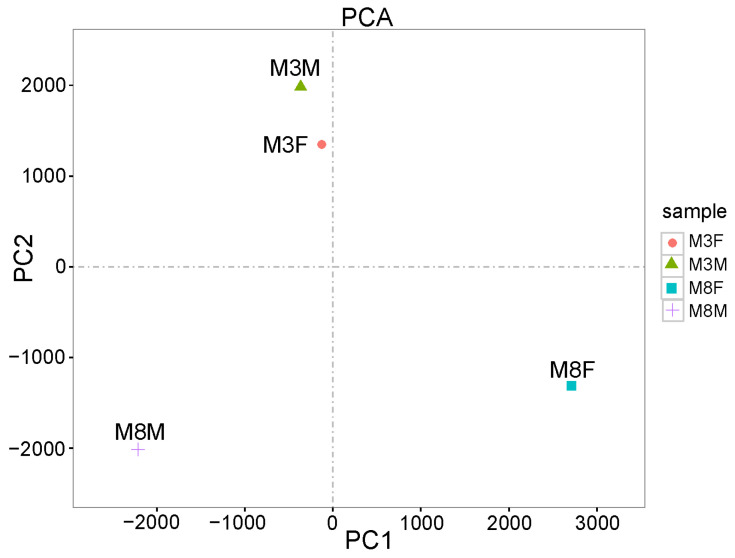
PCA analysis of M3M, M3F, M8M, and M8F samples.

A Principal Components Analysis (PCA) was performed on four sample groups based on methylation rate information at CG base sites (Figure 5). The PCA results showed that the M3M and M3F groups clustered well together, while the M8F and M8M groups were relatively scattered. This indicated that the difference between the M3M and M3F groups was minimal, whereas the difference between the M8F and M8M groups was more pronounced.

### 2.5. Enrichment Analysis of Differentially Methylated Sites (DMCs) and Associated Genes

**Figure 6:**
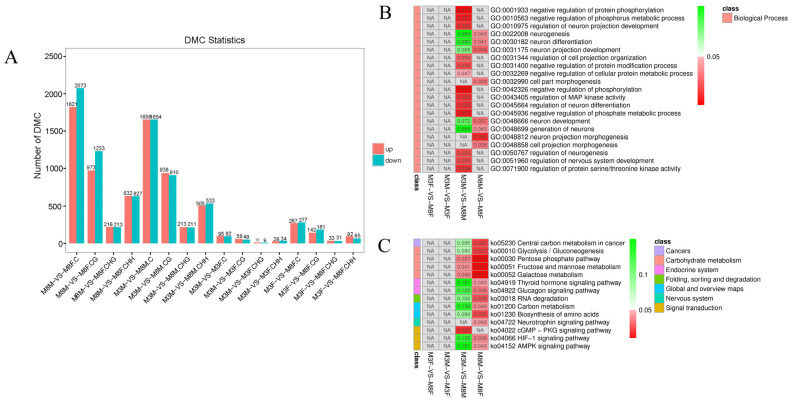
Enrichment analysis of differentially methylated sites (DMCs) and associated genes: (**A**) Genome-wide analysis of DMCs; (**B**) GO functional analysis of genes associated with DMCs; (**C**) Enrichment analysis of KEGG signaling pathway for methylation site-related genes.

Among the identified sites, the most significant differences were observed between M8M and M8F Hezuo pigs. Counts of C, CG, CHG, and CHH sites were 3894, 2206, 429, and 1259, respectively. The counts of C, CG, CHG, and CHH sites for the comparison between M3M and M8M were 3310, 1848, 424, and 1038, resperctively. The smallest differences between M3M and M3F Hezuo pigs showed counts of 187, 108, 19, and 60, respectively (Figure 6A, Table S1).

Furthermore, GO and KEGG enrichment analyses of genes linked to DMCs revealed no significant differences between M3M and M3F Hezuo pigs or between M3F and M8F Hezuo pigs. Significant differences were found in protein serine/threonine kinase activity, MAP kinase activity regulation, nervous system development regulation, negative regulation of phosphorylation and phosphate metabolism, and negative regulation of protein phosphorylation and modification between M3M and M8M (*p* < 0.05). Significant differences were also observed in the negative regulation of protein phosphorylation, regulation of neuron projection development, regulation of AMP kinase activity, regulation of neurogenesis, and regulation of the nervous system development process between M3M and M8M (*p* < 0.05). Significant differences in neuron projection development and neuron development were found between M8M and M8F Hezuo pigs (Figure 6B, Table S2). The KEGG signaling pathways between M3F and M8F, and between M8M and M8F Hezuo pigs, were significantly enriched in the biosynthesis of amino acids, Neurotrophin signaling pathway, and AMPK signaling pathway, among others (Figure 6C, Table S3).

### 2.6. Enrichment Analysis of DMR and Associated Genes

**Figure 7:**
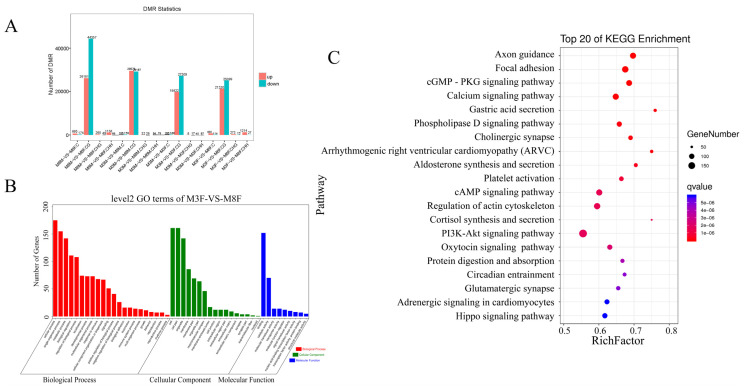
Enrichment analysis of differentially methylated regions (DMR) and associated genes: (**A**) DMR statistics; (**B**) GO functional; (**C**) KEGG enrichment analyses of signaling pathway in DMR-related genes.

The genome was scanned using a 200 bp window. In each window, the average DNA methylation rate for a specific cytosine type was calculated, and methylation levels were compared across samples. Analysis of the DMRs revealed CG as the most prevalent DMR type across different groups. Between M8M and M8F, 28,244 DMRs were significantly up-regulated, and 44,646 were down-regulated. Between 3-month-old and 8-month-old Hezuo pigs, 29,894 DMRs were up-regulated and 29,470 down-regulated. There were 23,488 significantly up-regulated DMRs and 25,252 significantly down-regulated DMRs between 3-month-old and 8-month-old Hezuo pigs. There were 20,050 significantly up-regulated and 27,579 significantly down-regulated DMRs between M3M and M3F Hezuo pigs (Figure 7A, Table S4).

GO function and KEGG pathway annotation analyses were performed on selected DMR genes. DMR-related genes were primarily enriched in cell and metabolic processes, biological regulation, cellular components, and catalytic activities (Figure 7B, Table S5). Key enriched KEGG pathways include the Hippo, PI3K-Akt, adrenergic signaling in cardiomyocytes, and actin cytoskeleton regulation (Figure 7C, Table S6).

### 2.7. Transcriptome Sequencing Results

**Figure 8:**
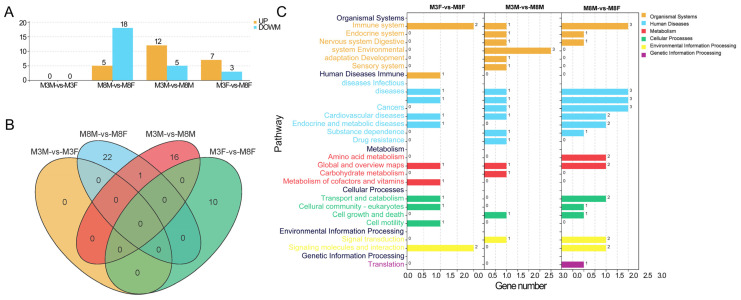
Transcriptome sequencing results: (**A**) Significantly differentially expressed genes; (**B**) Venn diagram-specific gene analysis; (**C**) Significant enrichment analysis of DEGs in the KEGG signaling pathway.

RNA-seq results indicated no significant DEGs between M3M and M3F Hezuo pigs. For M8M and M8F Hezuo pigs, there were 5 significantly up-regulated and 18 down-regulated genes. Between M3M and M8M, 12 genes were significantly up-regulated, and 5 were down-regulated. Between M3F and M8F, seven genes were up-regulated, and three were down-regulated (Figure 8A, Table S7). The Venn diagram analysis revealed 16, 10, and 22 specific genes for M3M vs. M8M, M3F vs. M8F, and M8M vs. M8F, respectively (Figure 8B). These genes were significantly enriched in KEGG pathways related to the immune and digestive systems and signal molecule interactions (Figure 8C).

### 2.8. Screening for DMGs Associated with Adult Weight Development in Hezuo Pigs

**Figure 9:**
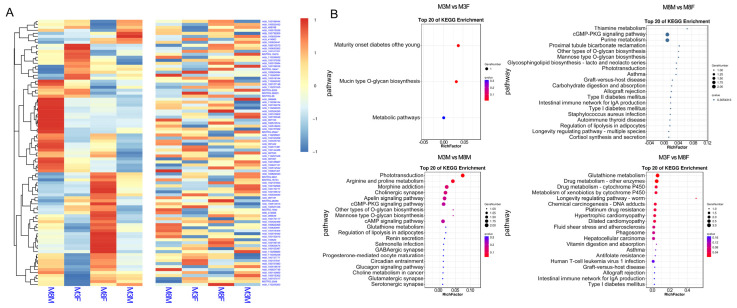
Screening of key methylation regulation genes for adult body weight differences: (**A**) Cluster heat map of DMR and DEG common gene expression level; (**B**) KEGG analysis results of DMR and DEG common genes.

To explore the relationship between methylation degree and gene expression, we performed a joint analysis of methylation and transcriptome data. From comparative analyses of DMR genes and DEGs across all groups, we identified common genes and clustered them (Figure 9A). A subsequent KEGG signal pathway enrichment analysis was conducted on these genes (Figure 9B). The gene annotation results revealed key genes involved in the growth hormone synthesis and secretion signaling pathway and the hypothalamus-pituitary-gonadal axis. We identified five candidate methylation genes (*CCL2*, *CTSH*, *MCH2*, *MYL2*, and *GST*) linked to adult body weight in Hezuo pigs. All of them are present in the promoter region of the gene (Table 3). These genes potentially influence the growth and development of boars and sows through the regulation of hormones associated with cAMP, PI3K-Akt signal transduction, and adrenergic signaling pathways.

### 2.9. Correlation Analysis of Different Hormones with Gene Expression

**Figure 10:**
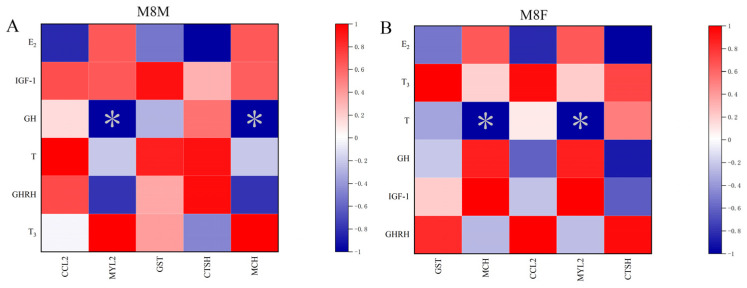
Correlation analysis of E2, GHRH, T, GH, IGF-1, and T3 with gene expression in Hezuo pigs (* indicates *p* < 0.05). (**A**) M8M and (**B**) M8F.

The Spearman’s correlation analysis conducted on candidate methylated genes related to adult body weight in the M8M and M8F groups showed significant negative correlations. GH in the M8M group and T in the M8F group were significantly negatively correlated with the expression levels of *MYL2* and *MCH* (*p* < 0.001), yielding a correlation coefficient of −1 (r = −1) as illustrated in Figure 10.

## 3. Discussion

In the growth and development of animals, the occurrence of larger females and smaller males is common. The disparity is important for survival, reproduction, and energy requirements. For example, females of some fish and invertebrates need larger ovaries and reproductive organs to produce and store more eggs, resulting in size disparity. In livestock production, the outcome equally depends on factors such as breed, production methods, and management goals. Hezuo pigs exhibit larger female and smaller male body weights in adulthood, which supports breeding objectives and enhances breeding strategies. Moreover, female Hezuo pigs require significant energy and nutrients during pregnancy and nursing. Thus, female Hezuo pigs tend to accumulate more body weight in adulthood to meet reproductive needs. However, the underlying phenotypic mechanisms for adult body weights differing between genders in Hezuo pigs remain unidentified. Thus, we examined two growth stages (3 and 8 months) in Hezuo pigs, initially measuring hormones E2, GHRH, GH, T, T3, and IGF-1 and then conducting a DNA methylation analysis to explore the epigenetic basis for their size differences.

The primary function of the pituitary is to regulate postnatal growth in animals [13,14]. The growth-promoting effects of the pituitary gland depend on GH [15], which enhances protein synthesis and metabolism either directly through its receptors or indirectly by stimulating IGF-1. GH deficiency results in growth retardation, delayed skeletal maturation, and weight loss [16]. GHRH stimulates GH secretion and synthesis and is thought to lead to GH proliferation [17]. Research indicates that anterior pituitary hypopituitarism may stem from GHRH resistance [18]. Gene deletion encoding GHRH led to a significant increase in lifespan in GH-deficient mice [19,20,21], underscoring the critical role of GHRH in GH secretion regulation. IGF-I regulates GH function and controls aging and longevity. In organisms with GH or GH receptor defects, reduced the binding of GH and IGF-I, along with insulin signaling, extends lifespans [19], offering new insights into aging mechanisms. This study found significantly higher serum GH levels in females than in males and significantly higher IGF-I levels in females than in males in 3-month-old Hezuo pigs. Conversely, serum GH levels were significantly lower in males than in females in 8-month-old Hezuo pigs, suggesting sex-dependent GH secretion stages that reduce IGF-1 secretion and stimulate skeletal muscle growth. T3, a biologically active thyroid hormone, regulates energy balance and boosts basal metabolic rate [22,23], playing a vital role in growth maintenance [24]. E2, a bio-endogenous natural estrogen, adversely affects growth [25]. High T concentrations induce negative feedback on the hypothalamus and pituitary gland, reducing hormone secretion, and T binding stimulates IGF-I secretion [26]. This study found higher serum T levels in females; however, 8-month-old Hezuo pigs showed lower T3 and E2 levels in females than in males and lower T levels in females than in males. Existing research indicates that E2 plays a role in inhibiting weight gain in mice through an enhancement of energy expenditure [26]. The hypothesis is that reduced E2 secretion in M8F Hezuo pigs, due to decreased ovarian function, leads to lower metabolism and weight gain. Furthermore, extremely lower serum T levels in females at 8 months suggest that T binding promotes GH secretion by slowing IGF-I stimulation, resulting in faster weight gain in females.

This study compared DNA expression patterns in the pituitary tissues of Hezuo pigs at different ages using sulfite sequencing. We explored the regulatory role of DNA methylation in the developmental processes of these pigs. The PCA revealed that the sample distributions of M3M and M3F Hezuo pigs were concentrated with consistent methylation trends. However, at 8 months, the distributions were scattered, highlighting physiological differences between the genders. This suggests that DNA methylation changes may regulate pig growth and development. The methylation patterns showed CG methylation as the predominant type in genomic DNA. Within the CG sequence environment, the highest methylation level was in the gene coding region, aligning with patterns observed in mammals like mice [27]. This indicates a conserved DNA methylation pattern across different gene regions.

To accurately assess genomic methylation, DEGs were analyzed. The statistical analysis of DMRs revealed CG as the most common type of DMRs among groups. The most significant differences in DMRs were between M8M and M8F pigs. The analysis of these regions showed more hypermethylated and hypomethylated areas in 8-month-old pigs. The higher proportion of hypomethylated regions suggests they may significantly influence the size differences between female and male Hezuo pigs.

We conducted GO and KEGG analyses on the identified DMR genes, identifying signaling pathways such as PI3K-Akt, Hippo signaling pathway, and adrenergic signaling pathways concerning muscle growth and development, hormone secretion and synthesis, and additional biological processes related to body weights of adult animals. This indicates that methylation regulation is crucial in the growth and development of Hezuo pigs, activating significant genes and pathways related to growth and metabolism. The PI3K-Akt signaling pathway regulates muscle growth and development, homeostasis maintenance, and other vital biological processes [28]. These insights offer robust theoretical support for our detailed exploration of the molecular mechanisms regulating adult body weight. Our results can inspire new avenues for understanding the molecular regulatory mechanisms behind the differences in adult body weight between male and female Hezuo pigs.

The integrated analysis of methylation and transcriptome data revealed diminished methylation levels in the promoter regions for the high-expression cohort, whereas increased methylation intensity was observed in the low-expression cohort. This indicates a strong negative correlation between methylation degree and gene expression near these regions. Current studies confirm that DNA methylation is generally negatively correlated with gene expression. Hypermethylation in the promoter regions inversely affects gene expression. A weak negative correlation exists between methylation within genes and gene expression; conversely, hypomethylation in promoter regions correlates positively with transcriptional activity [29]. Five genes, *CCL2*, *CTSH*, *MCH2*, *MYL2*, and *GST*, were identified. *CCL2* can activate chemotactic mononuclear macrophages and microglia and is a potent immune regulatory chemokine [30,31]. A deficiency in *CCL2* can hasten cognitive dysfunction by reducing neurogenesis and differentiation in the hippocampal gyrus [32]. *MYL2* influences muscle fiber activity and regulates muscle fiber types through ATPase activity on myosin-heavy chains, impacting muscle growth and development [33,34]. The *MYL2* gene, more expressed in Duroc and Yorkshire pigs than in Brazilian native Piau pigs, is involved in muscle development signaling pathways [35]. *GST* protects DNA from damage and aids in DNA repair [36]. *CTSH*, a key endopeptidase in the cathepsin family, is crucial for protein degradation in lysosomes [37]. *MCH* is a significant pathogen; knockout mouse models have shown MCH’s role in regulating energy homeostasis. Animals lacking *MCH* or its receptors resist diet-induced obesity [38]. It is suggested that candidate methylated genes related to adult body weight significantly influence animal growth and development.

The analysis of candidate methylated genes related to adult body weight in Hezuo pigs from the M8M and M8F groups revealed a significant negative correlation with hormone levels: GH in M8M and T in M8F negatively correlated with *MYL2* and *MCH* expression. Although each group showed a relatively high correlation, an ideal analysis would involve pituitary biopsies from all developmental stages of the same pig to eliminate individual differences. It has been shown that genes regulate organisms by controlling hormone secretion and metabolism [39]. A study on the ovaries of Hu sheep with different fecundity and genotypes through Whole-Genome Bisulfite Sequencing (WGBS) and RNA sequencing (RNA-seq) revealed that DNA methylation affects fecundity by influencing gene expression in the ovaries [40]. Studies have also found that DNA methylation plays a pivotal role in the response of Bombyx mori to environmental stress [41], providing crucial clues for identifying key resistance genes in the silkworm’s response to high temperature/humidity stress. These studies demonstrate a significant correlation between DNA methylation patterns and growth-related phenotypes. Hence, we hypothesized that methylated genes regulating adult body weight in Hezuo pigs might influence their growth by modulating the secretion and synthesis of related hormones, ultimately impacting adult body weight.

## 4. Materials and Methods

### 4.1. Experimental Animal Selection

In this study, 12 litters of healthy Hezuo pigs were divided into four groups. Each group consisted of three pigs selected from three different litters: 3-month-old male group (M3M, 6.01 ± 0.164 kg), 3-month-old female group (M3F, 6.45 ± 0.224 kg), 8-month-old male group (M8M, 13.75 ± 0.791 kg), and 8-month-old female group (M8F, 16.08 ± 0.414 kg). The Hezuo pigs, semi-grazing in agro-pastoral areas, grazed during the day, foraging on plant roots and tubers, and received supplementary soybean dregs at night. All boars remained uncastrated. Blood from the anterior vena cava was collected from three Hezuo pigs in each group at 3 and 8 months. The serum was separated for analyzing growth hormone dynamics. Three pigs from each group were euthanized via the injection of pentobarbital sodium solution. Pituitary tissue samples were immediately frozen in liquid nitrogen and stored at −80 °C. Following the manufacturer’s protocol, genomic DNA was isolated using a TIANamp Genomic DNA Kit (Tiangen, Beijing, China). DNA integrity and quality were assessed using agarose gel electrophoresis and spectrophotometry with a NanoDrop spectrophotometer (IMPLEN, Westlake Village, CA, USA).

### 4.2. Detection of Serum Hormone Content

An enzyme-linked immunosorbent assay (ELISA) was used to measure GH, growth hormone-releasing hormone (GHRH), IGF-1, triiodothyronine (T3), estradiol (E2), and testosterone (T) levels in Hezuo pigs at various developmental stages.

### 4.3. DNA Bisulfite Treatment and Sequencing

DNA samples from the pituitary tissue of Hezuo pigs were extracted and assessed for quality. A Pool-GWAS was performed using the EZ DNA Methylation Direct Kit (Zymo Research Corporation, Irvine, CA, USA). The average genomic DNA fragment size from pituitary tissues was about 150 bp, measured using a Covaris S220 ultrasonograph (Covaris, Woburn, MA, USA). After fragmentation, DNA fragments with 3′-end depressions were extracted with dA and evaluated for quality. Following A repair, the ends were blunted. Adapters were added following the manufacturer’s instructions, and the adapter-modified DNA was subjected to the sodium bisulfate reaction. Bisulfite-treated DNA was PCR amplified and subjected to paired-end sequencing on the Illumina HiSeq 2500 system (Illumina, San Diego, CA, USA). Sequencing reads were 150 nucleotides long.

### 4.4. Methylation Level Analysis

The clean reads were mapped to the pig reference genome using the BSMAP software [42] (version: 2.90) by default. A custom Perl script named methylated cytosines, and these cytosines underwent testing with the correction algorithm from Lister et al. [43]. Methylation levels were calculated as the percentage of methylated cytosines against the total in each sequence context (CG, CHG, CHH) across the whole genome, individual chromosomes, and various genome regions. To evaluate methylation patterns across different genomic areas, methylation maps for flanking 2 kb regions and gene bodies (or transposable elements) were created, using the average methylation level for each window. This approach was repeated to assess methylation differences in various genomic areas.

### 4.5. Detection and Analysis of Methylation Sites

In this study, only methylation sites with a sequencing coverage depth exceeding one were considered. Based on the data, the methylation rate at the C site was calculated using the following formula: [methylated reads/(methylated reads + unmethylated reads)] × 100% [44]. This formula accurately reflects the methylation status of the C base, providing a foundation for subsequent data analysis. Additionally, the genome-wide methylation level was calculated to better understand the overall characteristics of the genome methylation map and to ensure data accuracy and reliability. These calculations and screening steps yielded high-quality methylation data, supporting subsequent analyses of methylation differences and functional annotation.

### 4.6. Analysis of DMRs

To identify DMRs among M3M, M3F, M8M, and M8F Hezuo pigs, a minimum read coverage of four was required for calling base methylation status. DMRs for each sequence (CG, CHG, and CHH) were determined based on different scoring criteria: (1) For CG, the number of GCs in each window ≥ 5, absolute difference in methylation ratio ≥ 0.25, and q ≤ 0.05; (2) For CHG, the number in a window ≥ 5, absolute difference in methylation ratio ≥ 0.25, and q ≤ 0.05; (3) For CHH, the number in a window ≥ 15, absolute difference in methylation ratio ≥ 0.15, and q ≤ 0.05; (4) For all Cs, the number in a window ≥ 20, absolute difference in methylation ratio ≥ 0.2, and q ≤ 0.05.

### 4.7. Functional Enrichment Analysis of Differentially Methylated Site (DMC)/DMR-Related Genes

To analyze the functional enrichment of genes affected by DMCs/DMRs, gene ontology (GO) and KEGG pathway enrichment analyses were conducted on DMC/DMR-related genes. The GO enrichment analysis provides all GO terms that significantly enriched in genes compared to the genome background and filters the genes that correspond to biological functions. Firstly, all ceRNAs were mapped to GO terms in the gene ontology database (http://www.geneontology.org/), gene numbers were calculated for every term, and significantly enriched GO terms in genes compared to the genome background were defined by hypergeometric tests. Genes usually interact with each other to play roles in certain biological functions. A pathway-based analysis helps to further understand genes biological functions. KEGG is the major public pathway-related database (http://www.kegg.jp/kegg/) (accessed on 6 June 2024). The pathway enrichment analysis identified significantly enriched metabolic pathways or signal transduction pathways in genes compared with the whole genome background.

### 4.8. Total RNA Extraction and RNA-Sequencing

Total RNA was extracted from the pituitary tissue of 12 Hezuo pigs using a Trizol kit (Invitrogen, Carlsbad, CA, USA). After extraction, eukaryotic mRNA was enriched with Oligo (dT) magnetic beads. Subsequently, rRNA was removed to enrich prokaryotic mRNA, which was then fragmented into small pieces and reverse transcribed into cDNA. The second-strand cDNA was synthesized using DNA polymerase I, RNase H, dNTPs, and buffer. The cDNA fragments were purified with a QiaQuick PCR extraction kit (Qiagen, Venlo, The Netherlands), prepared with end-repair poly (A) tailing, and then linked to an Illumina sequencing adapter. Illumina HiSeq 2500 (Illumina, San Diego, CA, USA) was employed for sequencing in Guangzhou, China. To ensure high-quality reads, filtering was performed using fastp (version 0.18) [45]. The RNA differential expression analysis was performed by DESeq2 [46] software between two different groups (and by edgeR [47] between two samples). The genes/transcripts with parameters of false discovery rate (FDR) below 0.05 and absolute fold change ≥ 2 were considered differentially expressed genes/transcripts.

### 4.9. Statistical Analysis

A *t*-test was used to assess the significance of hormone variations in Hezuo pigs across M3M, M3F, M8M, and M8F groups using SPSS 26.0 (SPSS, Chicago, IL, USA). Data are presented as mean ± standard error. A *p*-value < 0.05 indicated a significant difference, while a *p*-value < 0.01 indicated a highly significant difference. The Spearman’s correlation analysis evaluated the relationship between various hormones and methylation candidate genes in M8M and M8F groups of Hezuo pigs using Origin 2022 (OriginLab, Waltham, MA, USA). Data with *p* < 0.05 were considered significant, and *p* < 0.01 were deemed highly significant.

## 5. Conclusions

This study analyzed the DNA methylation mechanisms influencing adult body weight in Hezuo pigs. Variations in serum T, E2, and T3 hormones at different developmental stages might correlate with adult body weight differences. DNA methylation could affect Hezuo pig body weight by altering the expression of genes related to growth and development. This study offers new insights into the regulatory mechanisms of DNA methylation in the weight gain process of Hezuo pigs and serves as a reference for studying animal sex traits.

## Figures and Tables

**Table 1:** Sequencing data quality.

Sample	Total Reads	Mapped Reads	Mapped Ratio (%)	Genome Size	Sequence Depth
M3M	455,956,276	378,242,478	82.96	2,485,970,876	22.82
M3F	481,350,952	401,708,691	83.45	2,485,970,876	24.24
M8M	465,351,434	386,271,639	83.01	2,485,970,876	23.31
M8F	468,237,436	390,204,927	83.33	2,485,970,876	23.54

**Table 2:** Genome-wide methylation level of methylated C (mC) locus in M3M, M3F, M8M, and M8F Hezuo pigs.

Sample	C (%)	mCG (%)	mCHG (%)	mCHH (%)
M3M	5.15	76.25	1.03	1.15
M3F	4.92	76.16	0.94	1.05
M8M	4.97	77.04	0.99	1.10
M8F	4.76	77.13	0.98	1.08

**Table 3:** Positional information on candidate methylated genes.

Gene ID	Symbol	Chromosome	Genomic Context
LOC397422	*CCL2*	NC_010454.4	promoter
LOC396969	*CTSH*	NC_010449.5	promoter
LOC733682	*MCH2*	NC_010443.5	promoter
LOC396690	*MYL2*	NC_010456.5	promoter
LOC100152951	*GST*	NC_010449.5	promoter

## Data Availability

Data generated in this study has been deposited in the CNCB GSA database under the accession numbers PRJCA028302 (https://www.cncb.ac.cn/search/specific?dbId=bioproject&q=CRA017856) (accessed on 6 June 2024) and PRJCA028308 (https://ngdc.cncb.ac.cn/search/specific?db=bioproject&q=CRA017857) (accessed on 12 July 2024).

## Supplementary Materials

The following supporting information can be downloaded at: https://www.mdpi.com/article/10.3390/ijms252111488/s1.
